# Nitric oxide compounds have different effects profiles on human articular chondrocyte metabolism

**DOI:** 10.1186/ar4295

**Published:** 2013-09-11

**Authors:** María C de Andrés, Emilia Maneiro, Miguel A Martín, Joaquín Arenas, Francisco J Blanco

**Affiliations:** 1 INIBIC-Complejo Hospitalario Universitario A Coruña (CHUAC) Rheumatology Division, As Xubias 84, 15006-A Coruña, Spain; 2 Instituto de Investigación Hospital 12 de Octubre, "i+12", Madrid, Spain; 3 Red de Proteomica Proteo-Red/ISCIII, Madrid, Spain; 4 RETIC-RIER-ISCIII, Madrid, Spain

**Keywords:** chondrocytes, nitric oxide, apoptosis, mitochondria, glucose, osteoarthritis

## Abstract

**Introduction:**

The pathogenesis of osteoarthritis (OA) is characterized by the production of high amounts of nitric oxide (NO), as a consequence of up-regulation of chondrocyte-inducible nitric oxide synthase (*iNOS*) induced by inflammatory cytokines. NO donors represent a powerful tool for studying the role of NO in the cartilage *in vitro*. There is no consensus about NO effects on articular cartilage in part because the differences between the NO donors available. The aim of this work is to compare the metabolic profile of traditional and new generation NO donors to see which one points out the osteoarthritic process in the best way.

**Methods:**

Human healthy and OA chondrocytes were isolated from patients undergoing joint replacement surgery, and primary cultured. Cells were stimulated with NO donors (NOC-12 or SNP). NO production was evaluated by the Griess method, and apoptosis was quantified by flow cytometry. Mitochondrial function was evaluated by analysing respiratory chain enzyme complexes, citrate synthase (CS) activities by enzymatic assay, mitochondrial membrane potential (Δψm) by JC-1 using flow cytometry, and ATP levels were measured by luminescence assays. Glucose transport was measured as the uptake of 2-deoxy-[^3^H]glucose (2-[^3^H]DG). Statistical analysis was performed using the Mann-Whitney U test.

**Results:**

NOC-12 liberates approximately ten times more NO_2_^- ^than SNP, but the level of cell death induced was not as profound as that produced by SNP. Normal articular chondrocytes stimulated with NOC-12 had reduced activity from complexes I, III y IV, and the mitochondrial mass was increased in these cells. Deleterious effects on ΔΨm and ATP levels were more profound with SNP, and this NO donor was able to reduce 2-[^3^H]DG levels. Both NO donors had opposite effects on lactate release, SNP diminished the levels and NOC-12 lead to lactate accumulation. OA chondrocytes incorporate significantly more 2-[^3^H]DG than healthy cells.

**Conclusions:**

These findings suggest that the new generation donors, specifically NOC-12, mimic the OA metabolic process much better than SNP. Previous results using SNP have to be considered prudently since most of the effects observed can be induced by the interactions of secondary products of NO.

## Introduction

Articular cartilage is an avascular, non-insulin-sensitive tissue that utilizes glucose as the main energy source and as a precursor for glycosaminoglycan synthesis and a regulator of gene expression. Degradation of articular cartilage is a hallmark of osteoarthritis (OA) [1] and is associated with aberrant glucose metabolism [2,3]. On the other hand, the pathogenesis of OA is characterized by the production of high amounts of nitric oxide (NO), consequence of up-regulation of chondrocyte-inducible nitric oxide synthase (*iNOS*) induced by inflammatory cytokines, such as IL-1β and TNFα, and other factors [4-7].

Although it has been reported that NO causes chondrocyte apoptosis [8-10], production of high levels of endogenous NO by over-expression of the *iNOS *gene in transfected chondrocytes has not been found to cause cell death [11]. Other reports have proposed NO to be a physiologic regulator of mitochondrial respiration in chondrocytes [10,12,13]. A variety of NO donors have been demonstrated to suppress energy production by mitochondrial respiration in different cell types [14,15], an effect enhanced at low oxygen tensions [16], and firstly reported in chondrocytes by Johnston and collaborators [12].

Chondrocytes are highly glycolytic resident cells of articular cartilage that metabolize glucose as a primary substrate for ATP production [17]. However, oxygen does diffuse into articular cartilage and articular chondrocytes possess mitochondria and respire *in vivo *[18]. The superficial and middle zones of articular cartilage are not anoxic [19], and in this context, mitochondrial oxidative phosphorylation (OXPHOS) is 18 times as efficient in ATP generation as is glycolysis [14,20]. Furthermore, OXPHOS may account for up to one fourth of total steady-state ATP production within articular cartilage, and possibly more under conditions of increased energy demands associated with cartilage stress [21]. Besides this, mitochondria are important in regulating both caspase-dependent and caspase-independent apoptotic pathways [22-26].

It is generally accepted that the quantities of available oxygen and glucose can fluctuate considerably in connective tissues such as articular cartilage, growth plates and the intervertebral disc [27-29]. Articular chondrocytes consume less oxygen in comparison with most other cell types [30]. Consequently, anaerobic glycolysis forms the principal source of cellular ATP in cartilage.

The direct investigation of the function of exogenous NO production on articular chondrocytes has been hampered by the lack of uniformity between the different types of NO donor compounds [31]. Since the past decade, the diazeniumdiolates began to replace traditional donors, such as SIN-1 (3-morpholinosydnonimine), SNP (sodium nitroprusside), SNAP (S-nitroso-N-acetylpenicillamine) and S-nitrosogluthathione, as sources of exogenous NO production [32], because have been shown to be reliable sources of NO under a variety of culture conditions [33,34]. The main advantages of these compounds are: known rates of NO generation, NO generation rates covering a wide range, spontaneity of NO generation and tenable generation of NO redox forms [34].

For all these reasons, besides the classical donor SNP, we used a diazeniumdiolate, NOC-12 (N-ethyl-2(1-ethyl-2 hydroxy-2-nitrosohydrazine), with a half-life of 327 minutes (determined at 22°C, pH 7.4) [33], for exogenous NO production, to further investigate the conditions in which NO is cytotoxic to chondrocytes, and compare the different effects induced by the two different types of NO donors. We wished to determine whether NO modulates the pathogenesis of OA, inducing apoptosis by means of the inhibition of mitochondrial function

## Materials and methods

### Cartilage acquisition and cell isolation

Normal human cartilage from femoral heads (joint replacement surgery) and knees (joint replacement surgery and autopsies) was obtained from 11 adult donors without history of joint disease and who had macroscopically normal cartilage (mean age ± SD 46.5 ± 10.5 years); human OA cartilage was obtained from the femoral heads of 12 patients (age 70 ± 12.6 years). All patients and healthy donors have signed the informed consent and the project was approved by the Regional Ethical Committee from Galicia (Spain). Small cartilage fragments were digested as previously described [35].

### Primary culture of chondrocytes

Chondrocytes were recovered and plated at high density (4 × 10^6 ^per 162 cm^2 ^flask; Costar, Cambridge, MA, USA) in DMEM (Life Technologies, Paisley, UK) supplemented with 100 units/ml penicillin, 100 μg/ml streptomycin, 1% glutamine, and 10% FCS (Life Technologies). Chondrocytes were incubated at 37°C in a humidified gas mixture containing 5% CO_2 _balanced with air. Chondrocytes were used at weeks 2 to 3 at confluence in primary culture. Cell viability was assessed by trypan blue dye exclusion, and stained cells were discarded to carry out experiments.

### General procedure and NO donor compounds employed

NO donor compounds were added from 60 mM stock solution dissolved in 0.1 M NaOH (NOC-12) and 10 mM stock solution dissolved in medium (SNP). This NO donor compound was freshly prepared before each experiment but NOC-12 was stored as 60 mM stock solutions in 0.1 mM NaOH at -20°C. Chondrocytes were first seeded in DMEM with 5% FCS inactivated for 24 hours, and then the NO donor compound was added directly to the culture medium and allowed to incubate for an additional 5-, 12-, 24- and 48-hour period, depending on each experiment. Experiments without glucose were carried out in DMEM glucose-free medium (Life Technologies) supplemented in the same way as the standard.

### Quantification of nitrites

The NO production of chondrocyte cells was measured by estimating nitrite accumulation using the Griess reagent (1% sulphanilamide and 0.1% N-(1-naphthyl)-ethylenediamine dihydrochloride in 5% H_3_PO_4_) (Sigma, St Louis, MO) as previously described [36]. Chondrocytes were cultured in 96-well plates (5 × 10^4^) and stimulated with different NO donors for 5, 24 and 48 hours.

### DNA labelling technique with propidium iodide for flow cytometry analysis

Chondrocytes (500,000 cells/well in a 6-well plate) were incubated with different NO donors (0.5, 1.0 and 2.0 mM SNP and NOC-12) for 12, 24 and 48 hours. Then cells were fixed in 70% ethanol at 4°C for 60 minutes, washed and incubated with RNAse (50 μg/ml) and propidium iodide (PI, 100 μg/ml) for 15 minutes at room temperature in the dark and kept at 4°C. PI fluorescence of nuclei was measured by flow cytometry on a FACScan (Becton and Dickinson, Mountain View, CA, USA) using a 560-nm dichromatic mirror and a 600-nm band pass filter. Data are expressed as percent apoptotic (hypodiploid) nuclei.

### Morphological evidence of apoptosis

For morphological studies, chondrocytes were cultured in 8-well slides (Costar) and treated with 1 mM of different NO donors for 24 hours. The cells were then washed with cold PBS, fixed in acetone/ethanol 70% (1:1) for 10 minutes at 4°C, stained with 49,6-dianidino-2-phenylindole dihydrochloride (DAPI, 2 mg/ml) (Sigma) for 10 minutes in the dark, mounted in glycergel (DAKO, Hamburg, Germany), and observed by fluorescence microscopy (Olympus, BX-UCB, Center Valley, PA).

### Measurement of the MRC complex activities in digitonin-permeabilized chondrocytes

Untreated, SNP-treated (5 hours) and NOC-12-treated (24 hours) chondrocytes (at least 10 × 10^6^) were collected by trypsinization, washed with PBS, and sedimented at 150 *g *for 5 minutes at 4°C. Digitonin-permeabilized chondrocyte homogenates (10 to 50 μl per ml of test volume) were used to measure the activities of the respiratory chain enzymes and citrate synthase (CS) in a DU-650 spectrophotometer (Beckman Instruments, Palo Alto, CA, USA) as previously described [35].

### Determination of mitochondrial membrane potential (ΔΨm)

To measure the ΔΨm of chondrocytes, the fluorescent probe JC-1 (5,5',6,6'- tetrachloro-1,1',3,3'-tetraethylbenzimidazole carbocyanide iodide) (Molecular Probes, Inc., Eugene, OR, USA) was used. JC-1 exists as a monomer at low values of ΔΨm (green fluorescence), whereas it forms aggregates at high ΔΨm (red fluorescence). Briefly, chondrocytes were cultured in 6-well plates (5 × 10^5^) and stimulated with different NO donors for 5, 12 and 24 hours; after that they were prepared as previously described [35].

### Assay of intracellular ATP

To assay intracellular ATP, we used a commercial biolumiscence kit (ATPLite, PerkinElmer Inc., Boston, MA, USA). Chondrocytes were cultured in 96-well plates (5 × 10^4^) and stimulated with different NO donors for 24 hours (100 μl); after this, 50 μl of lysis solution were added and mixed for 5 minutes; subsequently the enzymatic substrate (luciferase/luciferine) was added. The kit supplies a standard that gives reference values. Readers were carried out in a microbeta counter (PerkinElmer Inc.).

### 2-Deoxy-[^3^H] glucose uptake

To determinate the glucose uptake levels, normal chondrocytes were cultured in 24- well plates (Visiplate, PerkinElmer Inc.) at 2 × 10^5 ^cells per well in DMEM without glucose and 5% inactivated calf serum for 24 h at 37°C. Later, cells were stimulated with 10 μM of different donors for 24 hours at 37°C in DMEM without glucose and subsequently 10 μCi 2-deoxy-(^3^H)glucose (2-(^3^H)DG) was added to cells in DMEM without glucose for 0 minutes, 15 minutes and 1 hour at 37°C. Cells were washed with cold PBS pH 7.4; later, 50 μl of solvable (PerkinElmer Inc.) was added to lysate cells and mixed vigorously for 5 minutes. Lastly, 500 μl of scintillation liquid was added (Ultima Gold, PerkinElmer, Inc.) and mixed for 2 minutes; glucose uptake was estimated by means of a microbeta counter (MicroBeta TriLux (PerkinElmer, Inc.)).

### Quantification of lactic acid

Enzymatic determination of lactic acid in chondrocyte culture supernatants was performed using Lactate Reagent (Spinreact, Girona, Spain). Chondrocytes were cultured in 96-well plates (5 × 10^4^) and stimulated with different NO donors for 24 hours; 10 μl of supernatant were mixed with 10 μl of lactate reagent and incubated for 5 minutes at room temperature. The absorption was estimated by an automated plate reader (Ultrospec 1000, Amersham Pharmacia Biotech, Uppsala, Sweden) at 505 nm; this method is linear towards lactate values of 150 mg/dl.

### Data analysis

Data analysis was performed with SPSS software, version 12.05 (SPSS, Chicago, IL, USA). Results are expressed as the mean ± SD. Individual donors were studied in duplicate; cells from different donors were not pooled in any experiment. Comparisons between groups were carried out using the Mann-Whitney two-tailed *U*-test. *P*-values ≤ 0.05 were considered significant.

## Results

### NO release by different NO donors

**Figure 1:**
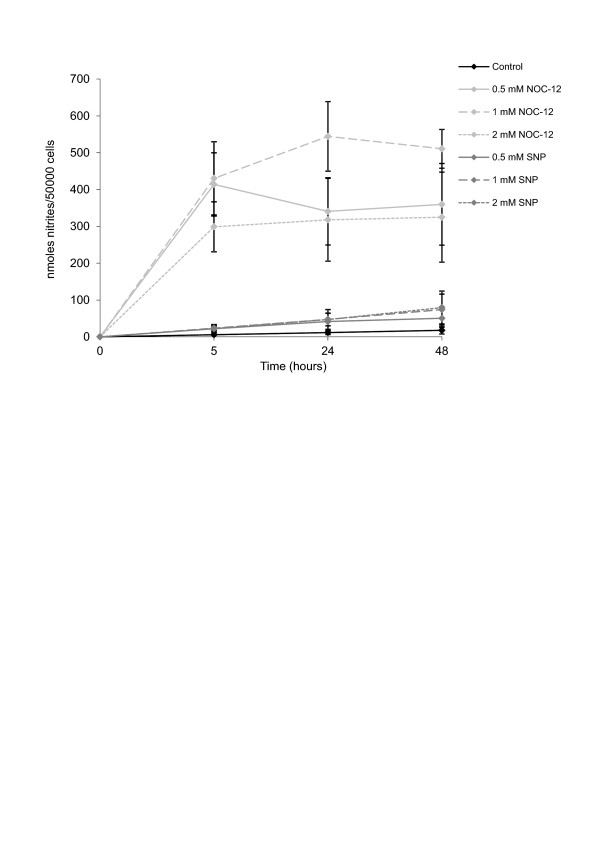
**Nitric oxide (NO) donors differ from the amount of NO_2_^- ^released**. NO_2_^- ^accumulation in supernatants of normal chondrocytes treated with different NO donors for 24 hours in standard culture conditions. The lines show the dose-dependent increase of NO accumulation in all cases. Values are the mean ± SD; *n *= 5. SNP, sodium nitroprusside; NOC-12, N-ethyl-2(1-ethyl-2 hydroxy-2-nitrosohydrazine.

We observed that kinetic liberation of NO changes between different NO donors. The diazeniumdiolate NOC-12 liberated approximately ten times more NO_2_^- ^than SNP. NO_2_^- ^accumulation in supernatants of normal chondrocytes treated with different NO donors depending on time and concentration (Figure 1).

### Effect of NO on cell death of normal human articular chondrocytes

**Figure 2:**
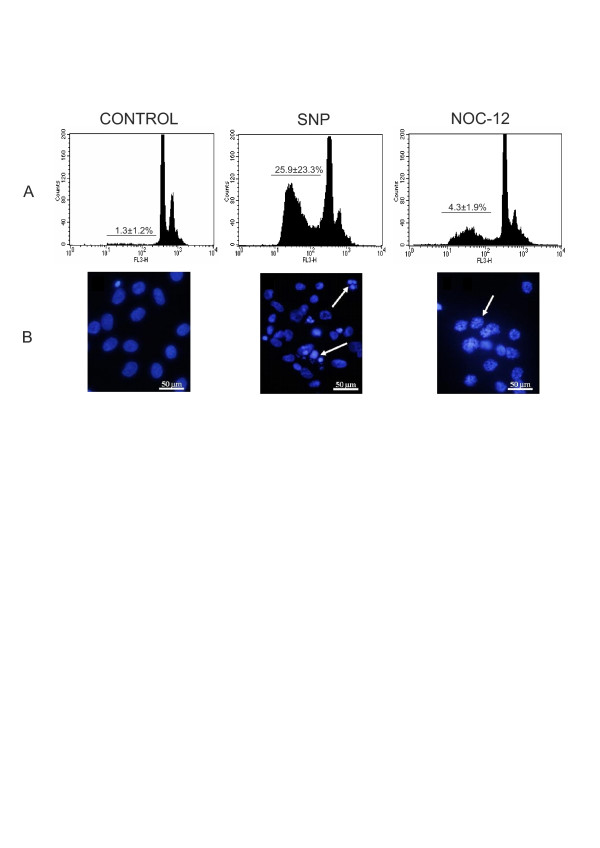
**Nitric oxide (NO) effect on chondrocyte apoptosis**. (**A**) Cell death levels in chondrocytes treated with different NO donors was determined by the iodide propidium (PI) method. Control chondrocytes were treated with 1 mM sodium nitroprusside (SNP) and 1 mM N-ethyl-2(1-ethyl-2 hydroxy-2-nitrosohydrazine (NOC-12) for 24 hours. Data are expressed as a percentage of apoptotic (hypodiploid) nuclei. Values are the mean ± SD; *n *= 5. *P ≤ 0.05 versus untreated chondrocytes (control). (**B**) Cellular changes induced by NO on normal human chondrocytes were analysed by 49,6-diamino-2-phenylindole dihydrochloride (DAPI staining) and fluorescence microscopy. Shown is a representative example of five experiments.

NO donors damage the nuclear DNA of human articular chondrocytes in very different ways. By means of flow cytometry with PI, we could observe that the percentage of death cells (expressed as hypodiplod DNA percentage) was much higher with SNP than NOC-12 (Additional file 1); with 1 mM SNP at 24 hours: 25.9 ± 23.3 versus 0.8 ± 0.5 (*P *≤ 0.05) compared with 1 mM NOC-12 at 24 hours: 4.3 ± 1.9 versus 0.8 ± 0.5 (*P *≤ 0.05) (Figure 2A). However, with higher concentrations of NOC-12 (2 mM at 48 hours), the percentage of apoptosis reached 21.6 ± 8.5% (Additional file 1). Besides this, when the cells were stained with DAPI we could see that the only NO donor able to induce the fragmentation of the nucleus and the formation of apoptotic bodies was SNP (Figure 2B). The only effect of NOC-12 on the nucleus of chondrocytes was the acquisition of a globule-like aspect (Figure 2B).

### NOC-12 alters the activity of the complexes of the mitochondrial respiratory chain (MRC) in articular chondrocytes

Previous results obtained in our laboratory showed that the activity of the complex IV is significantly lower in normal chondrocytes stimulated with 1 mM SNP at 5 hours than in control cells [10] (Table 1). In relation with the enzymatic activity of the MRC of normal chondrocytes treated with the diazeniumdiolate compound NOC-12, the activities of all complexes were significantly lower than in control cells, except complex II (Table 1). Enzyme activities were referred to the specific activity of CS to correct for mitochondrial volume.

**Table 1:** Values of mitochondrial respiratory chain complexes in cultures of normal chondrocytes treated with two NO donor compounds

	Normal chondrocytes	Cells treated with 1 mM NOC-12	Cells treated with 1 mM SNP
Age, years	49.8 ± 21.2 (14)	44.2 ± 19.4 (5)	59.7 ± 18.9 (11)
Proteins, mg/ml	3.1 ± 0.9 (14)	2.8 ± 1.4 (5)	4.2 ± 1.4 (11)
CS enzymatic activity (nmol/min/mg protein)	108.9 ± 38.5 (14)	146.9 ± 54.7 (5)	106.6 ± 26.2 (11)
Mitochondrial complex activity^a^			
Complex I	30.1 ± 13.4 (14)	6.8 ± 2.3 (5)*	22.8 ± 19.1 (11)
Complex II	7.4 ± 2.3 (14)	6.8 ± 1.6 (5)	10.2 ± 1.81 (11)
Complex III	53.5 ± 18.4 (14)	35.2 ± 9.3 (5)*	46.3 ± 9.7 (11)
Complex IV	61.2 ± 8.3 (14)	15.7 ± 3.1 (5)*	40.2 ± 11.3 (11)*

Values are the mean ± SD (number). *P ≤ 0.05 versus untreated chondrocytes. ^a^CS-corrected complex activity is expressed as (nmol/minute/mg protein)/(CS-specific activity) × 100. NO, nitric oxide; NOC-12, N-ethyl-2(1-ethyl-2 hydroxy-2-nitrosohydrazine; CS, citrate synthase; Complex I, rotenone-sensitive NADH-coenzime Q1 reductase; complex II, succinate dehydrogenase; complex III, antimycin-sensitive ubiquinol cytochrome c reductase; complex IV, cytochrome c oxidase. Chondrocytes were treated with NOC-12 for 24 hours and SNP for 5 hours.

### NO causes depolarization of the mitochondria in normal chondrocytes

**Figure 3:**
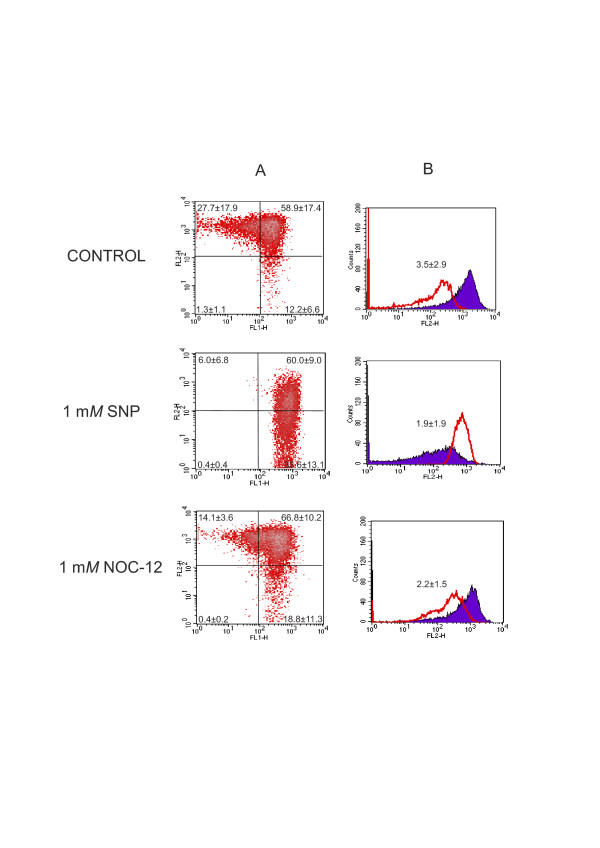
**Effect of nitric oxide (NO) donors on mitochondrial membrane potential**. (**A**) Fluorescence-activated cell sorter analysis of mitochondrial membrane potential in human chondrocytes. Untreated and treated normal chondrocytes with NO donors were stained with 5,5',6,6'- tetrachloro-1,1',3,3'-tetraethylbenzimidazole carbocyanide iodide (JC-1) and analysed by flow cytometry. Photomultiplier settings were adjusted to detect JC-1 monomer fluorescence signals on the filter 1 (FL1) detector (green fluorescence) and JC-1 aggregate fluorescence signals on the FL2 detector (red fluorescence). Shown is an example of chondrocytes treated with 1 mM SNP and 2 mM N-ethyl-2(1-ethyl-2 hydroxy-2-nitrosohydrazine (NOC-12) for 24 hours. (**B**) Quantification of red and green fluorescence. Histograms represent the JC-1 fluorescence of normal cells and those treated with NO donors. Green fluorescence (open graph) increases, whereas red fluorescence (solid graph) decreases in the NOC-12 and sodium nitroprusside (SNP)-treated chondrocytes, suggesting a reduction of the mitochondrial membrane potential, and therefore, a decrease in the red/green ratio. Shown is an example at 24 hours. Results are the mean ± SD; *n *= 5. *P ≤ 0.05 versus untreated chondrocytes (control).

The relative ratio of red/green fluorescence (ratio of normal mitochondrial polarization to mitochondrial depolarization) intensity values showed that in normal human chondrocyte cultures 1 mM SNP at 24 hours decreased the ratio of red/green fluorescence in comparison with untreated cells (1.91 ± 1.9 versus 3.5 ± 2.9; *P *≤ 0.01) (Figure 3B). In addition, 1 mM SNP caused an increase in the cell population with mitochondrial depolarization (12.2 ± 6.6 versus 33.6 ± 13.1%; *P *≤ 0.05) (Figure 3A). On the other hand, NOC-12 induced mitochondrial depolarization, as the percentage of cells with normal polarization diminished, with 1 mM NOC-12 at 24 hours: 27.7 ± 17.9 versus 14.1 ± 3.6; *P *≤ 0.05; (Figure 3A), and the percentage of cells with depolarization increased: 12.2 ± 6.6 versus 18.8 ± 11.3; *P *≤ 0.05 (Figure 3A). This finding also can be observed with decreasing ratio of red/green fluorescence in comparison with untreated cells (2.2 ± 1.5 versus 3.5 ± 2.9; *P *≤ 0.05) (Figure 3B). However, the NO donor that induced the strongest changes in the mitochondrial membrane potential was SNP.

### NO abolishes ATP generation by chondrocytes in culture

**Figure 4:**
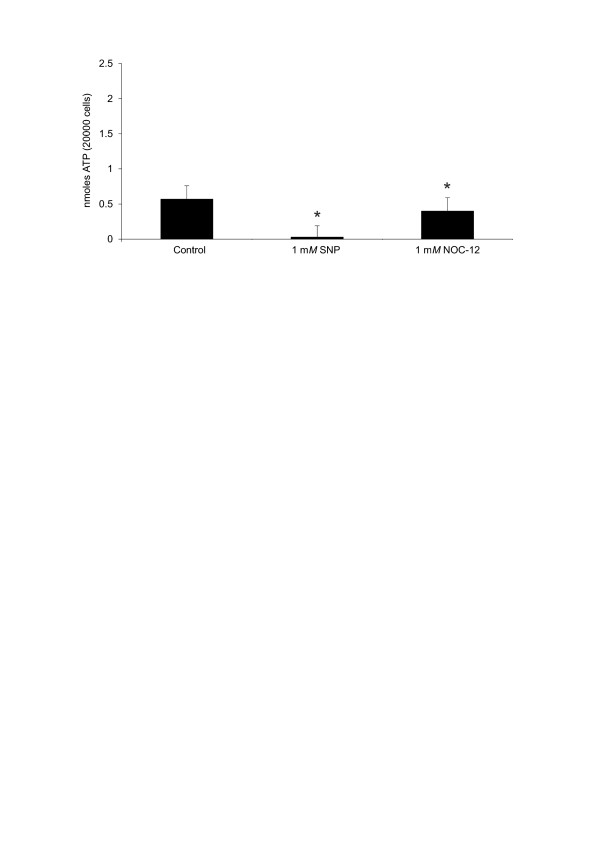
**ATP generation by normal chondrocytes treated with different nitric oxide (NO) donors**. Articular chondrocytes were treated with 1 mM sodium nitroprusside (SNP) and 2 mM N-ethyl-2(1-ethyl-2 hydroxy-2-nitrosohydrazine (NOC-12) for 24 hours. Then the ATP assay was performed as described in Materials and methods. Values are the mean ± SD; *n *= 7. *P ≤ 0.05 versus untreated chondrocytes (control).

NO has a detrimental effect on the generation of ATP by normal chondrocytes (Additional file 2). With NOC-12, the intracellular ATP levels were significantly lower than in control cells, with 1mM NOC-12 at 24 hours: 0.40 ± 0.16 versus 0.57 ± 0.19 (*P *≤ 0.05). Again, the NO donor that induced the most dramatic changes was SNP, as it reduced the intracellular ATP levels practically to zero (Figure 4).

### Chondrocytic lactate production is strongly influenced by NO donor type

NO donors have very different effects on lactate release by normal articular chondrocytes. The only donor that induced a significant fall in these values was SNP, with 2 mM SNP at 24 hours: 1.01 ± 0.3 versus 2.30 ± 0.58 (*P *≤ 0.05) (Table 2). The new generation donor NOC-12 induced a substantial increase in lactate production, with 0.5 mM NOC-12 at 24 hours: 2.91 ± 0.58 versus 2.30 ± 0.58, although this increase was not statistically significant (Table 2).

**Table 2:** Values of lactate release in cultures of normal chondrocytes treated with different NO donors compounds for 24 hours

	Lactate (μM/5 × 10^4 ^cells)
Control normal chondrocytes	2.30 ± 0.58 (7)
0.5 mM SNP	1.83 ± 0.65 (7)
1 mM SNP	1.33 ± 0.38 (7) †
2 mM SNP	1.01 ± 0.30 (7) †
0.5 mM NOC-12	2.91 ± 0.58 (7)
1 mM NOC-12	2.90 ± 0.66 (7)
2 mM NOC-12	2.86 ± 0.77 (7)

Values are the mean ± SD (number). NO, nitric oxide; SNP, sodium nitroprusside; NOC-12, N-ethyl-2(1-ethyl-2 hydroxy-2-nitrosohydrazine.

### SNP reduces glucose uptake by normal articular chondrocytes

**Figure 5:**
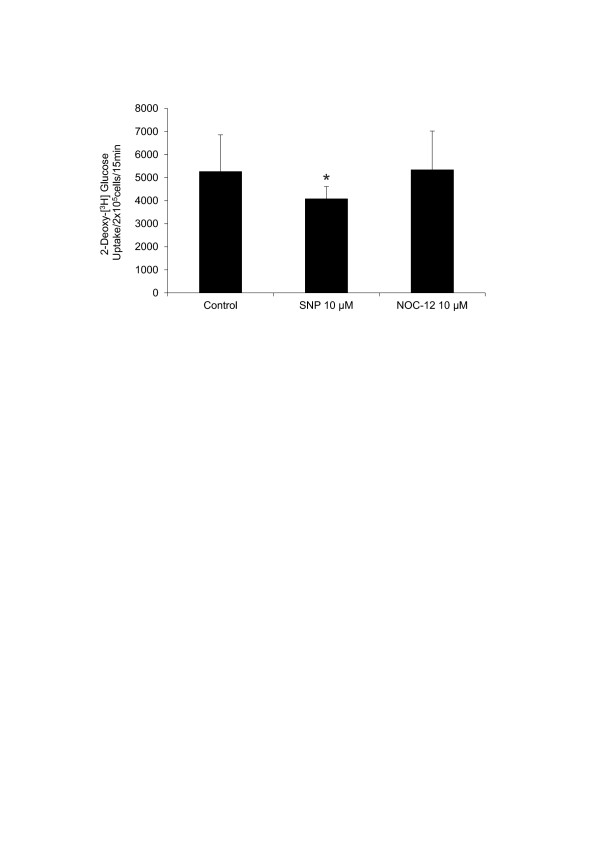
**Nitric oxide (NO) influence on 2-deoxy-(^3^H) glucose uptake by normal chondrocytes**. Effect of the NO donors sodium nitroprusside (SNP) and N-ethyl-2(1-ethyl-2 hydroxy-2-nitrosohydrazine (NOC-12) on the uptake of 2-deoxy-(^3^H) glucose by normal chondrocytes for 15 minutes. The detailed procedure is described in Materials and methods. Values are the mean ± SD; *n *= 7. *P ≤ 0.05 versus untreated chondrocytes (control).

Uptake of 2-(^3^H)DG by normal chondrocytes cultured under μM SNP concentrations (10 μM) for either 15 minutes or 1 hour was approximately 20% lower than that found in their respective controls (Figure 5). On the contrary, 2-(^3^H)DG uptake in normal chondrocytes stimulated with NOC-12 did not change relative to the control. In this set of experiments, μM NO donors concentrations were employed; the reason is because in most of these cases, when mM concentrations were used, this caused the cells to rise (above all, SNP) and the quantifications showed false positives.

### Detrimental effect of NO on chondrocyte viability depends on glucose levels

**Figure 6:**
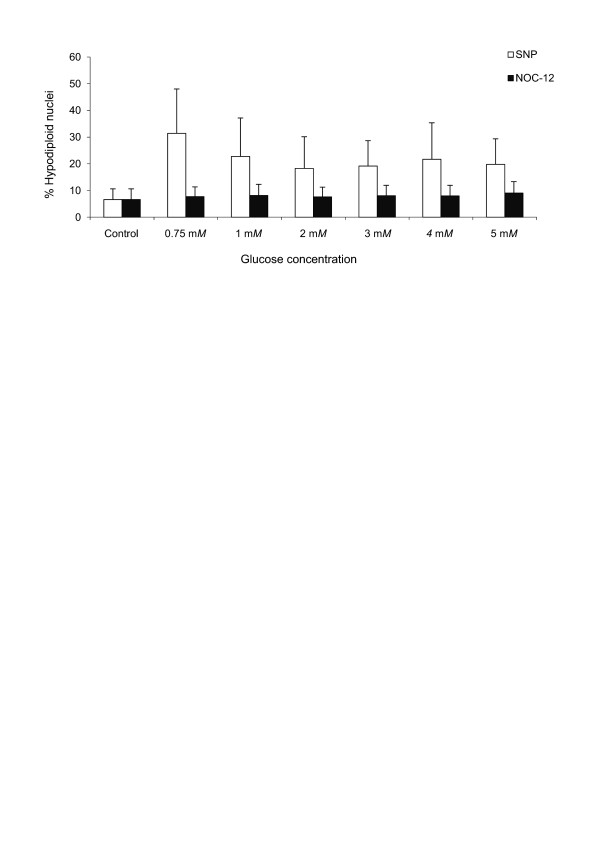
**The effect of nitric oxide (NO) on chondrocyte viability depends on glucose levels**. Articular chondrocytes were cultured in medium with crescent glucose concentrations and treated with 1 mM sodium nitroprusside (SNP) and 1 mM N-ethyl-2(1-ethyl-2 hydroxy-2-nitrosohydrazine (NOC-12) for 24 hours. Data are expressed as a percentage of apoptotic (hypodiploid) nuclei. Values are the mean ± SD; *n *= 3.

To assess the impact of glucose levels on cell viability after NO treatment, we carried out experiments with both NO donors (SNP and NOC-12) using only one constant concentration (1 mM) and increasing glucose concentrations (from 0.75 to 5 mM). The percentage of death cells decreased as glucose concentration increased, only when SNP was employed as NO donor (Figure 6). No significant results were found in this issue when NOC-12 was used.

### Glucose uptake by OA chondrocytes in basal conditions is more efficient than by normal chondrocytes

**Figure 7:**
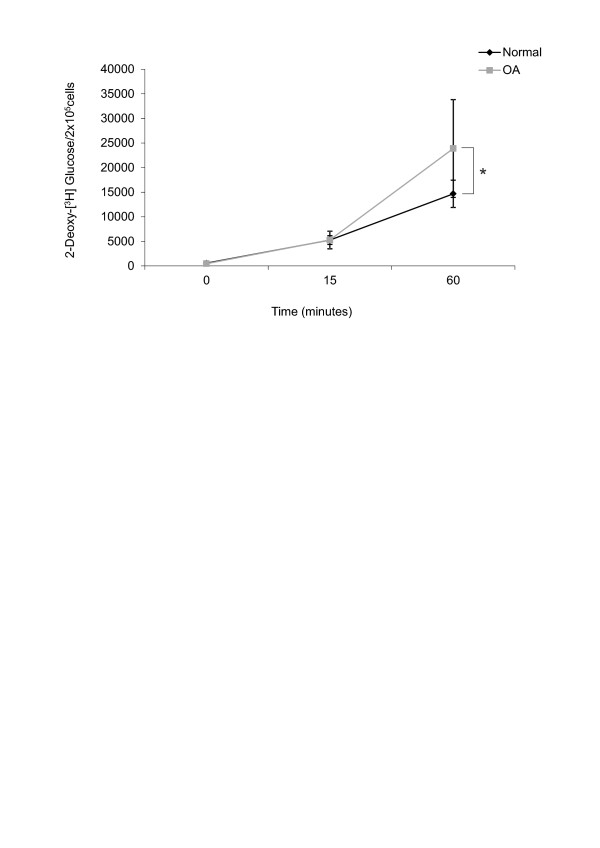
**Osteoarthritis (OA) chondrocytes are efficient cells in the uptake of glucose**. Differences between human articular normal and OA chondrocytes in the uptake of 2-deoxy-(^3^H)glucose for 1 hour. The detailed procedure is described in Materials and methods. Values are the mean ± SD; *n *= 12. *P ≤ 0.05 versus normal chondrocytes.

Chondrocytes were maintained for 15 minutes or 1 hour in culture media without glucose and a non-metabolizable analogue of glucose: 2-(^3^H)DG. Basal 2-(^3^H)DG uptake was identical in normal and OA chondrocytes incubated for 15 minutes; however, the basal 2-(^3^H)DG uptake in OA chondrocytes was significantly higher than in normal chondrocytes (23889.9 ± 9941.6 versus 14669.5 ± 2776.1; *P *≤ 0.05) (Figure 7), when cells were maintained for 1 hour in culture.

## Discussion

Traditionally, the increase of endogenous NO production by human articular cartilage has been associated with joint degeneration. NO donors have been used so far to mimic the OA process *in vitro*, and they represent a powerful tool of study. However, *in vitro *models with different NO donors have not resolved what the role of NO is in cartilage degradation due to the lack of uniformity that exists between the different types of NO compounds [31]. The differential effects of NO are partly due to the type of NO donors and cell used [37]. The biochemistry of NO is complex because of the reactions of NO itself, the interactions of secondary products of NO and the overall chemical environment under which NO is produced [38].

In our study, we employed two NO donor types: the traditional compound SNP, that is used in the majority of studies, and one diazeniumdiolate: NOC-12. It has been reported that the traditional donor SNP does not spontaneously release NO in the absence of redox activation [34]. Diazeniumdiolates, also denominated as NONOate, or NOC, have begun to replace traditional donors as sources of exogenous NO production [32] and have been shown to be reliable sources of NO under a variety of culture conditions [33,34]. For these reasons, the diazeniumdiolate compound NOC-12, with a half-life of 327 minutes (determined at 22°C, pH 7.4) [33] was used for exogenous NO production to further investigate the conditions in which NO is cytotoxic to chondrocytes. A primary basis for the use of diazeniumdiolates is that many of them decompose spontaneously in aqueous media to release the critical bioregulatory species [34]. The main advantages of these compounds are known rates of NO generation, NO generation rates covering a wide range, spontaneity of NO generation and tenable generation of NO redox forms.

The precise role of NO in the induction of chondrocyte death is repeatedly debated. Treatment with classical NO donors consistently induces apoptosis in cultured chondrocytes [6,8,10,39], whereas the production of high levels of endogenous NO by the over-expression of the *iNOS *gene in transfected chondrocytes has not been found to cause cell death [11]. This discrepancy might be the result of using chemical NO donors, which not only generate reactive nitrogen species but also produce various secondary reactions depending on the cellular milieu with *in vitro *experiments [40]. Also, an anti-apoptotic role has been addressed in several review articles [41-45]. Specifically, del Carlo and collaborators showed that compounds that only release NO, such as the diazeniumdiolates NOC-5 and NOC-12, do not cause chondrocyte cell death and can even be protective under certain conditions of oxidative stress [39]. It is likely that persistent spontaneous release of NO is necessary for the protective effect and that peroxynitrite and cyanide contribute to the cytotoxic effect of NO donors [37]. Chondrocyte cell death from NO occurs under conditions where other reactive oxygen species (ROS) are also generated [39].

Chondrocyte death does not correlate with the amount of NO released by NO donors. Similar to other authors [46], our results showed that SNP is the least potent in terms of producing exogenous NO in chondrocyte culture, although it is the most potent inducer of chondrocyte death. The amount of NO produced by NOC-12 was 10-fold higher than the NO produced by SNP, but the level of cell death induced was not as profound as that produced by SNP. As previously shown in our laboratory [10], SNP was able to induce formation of apoptotic bodies, which are produced from cells undergoing cell death by apoptosis. However, we observed that NOC-12 increased the hypodiploid nuclei number without formation of apoptotic bodies, which is probably related to another type of programmed cell death. Recently it has been proposed that autophagy is another type of programmed cell death than happens in the human articular cartilage as well [47]. The increase in the number of hypopliod nuclei and the observation of some morphologic changes as vacuole formation seems to relate NOC-12 with autophagy (personal data).

It is believed unlikely that NO is the sole mediator of SNP-induced chondrocyte death and peroxynitrite, a reaction product of NO and superoxide anions, or the primary by-products of the decomposition of SNP, such as the cyanide aninon or pentacy-anoferrate complex, might contribute to its cytotoxicity [34,48]. It is unclear whether chondrocyte apoptosis is the major mechanism of cartilage degradation or merely a by-product of tissue degeneration [46].

Mitochondria comprise a target of NO and there is accumulating evidence that inhibition of respiration may contribute to the pro-apoptotic effect of NO by ΔΨm alteration, transition-pore opening and release of cytochrome c [45,49]. There is increasing evidence about the importance of mitochondria in OA pathology. Previously, we showed that the activity of the mitochondrial complexes II and III is lower in OA than in normal human chondrocytes; this produces a decrease in ATP levels as well as a higher ROS generation [12,35]. The relevance of the MRC inhibition in human chondrocytes is already known, the inhibition of complexes III and V of the MRC induces an inflammatory response, which could be especially relevant in relation to prostaglandin E_2 _(PGE2) production via mitochondrial Ca^2+ ^exchange, ROS production, and nuclear factor (NF)-κB activation [50]. More recently, Rego and collaborators have found that the predisposition to the development of OA is related to some haplogroups of mitochondrial respiratory genes of chondrocytes [51]. Also, chondrocytes are not the only joint cells affected in OA pathology and, a recent study has shown that SNP reduces the survival of OA synoviocytes by regulating mitochondrial functionality, as well as the proteins controlling the cell cycle [52].

Analysis of the MRC showed that at 5 hours, SNP reduced the activity of complex IV by 30%; furthermore, SNP induced depolarisation of the mitochondrial membrane [10]. In this study we show that NOC-12 induces depolarisation of the mitochondrial membrane as well as SNP, but to a lesser extent (10%); however, it had a more radical effect on MRC activity than SNP, this donor reduces the activities of all the complexes except complex II (complex I by 80%, complex III by 33% and complex IV by 75%). These results show that the inhibition of the MRC complexes is not the main cause of cell death induction in chondrocytes by NO. On the other hand, CS activity was increased about 40% in NOC-12-treated chondrocytes, and this fact has been correlated with an increment of the mitochondrial mass [30]; Nisoli and collaborators also suggested that NO is implicated in the regulation of energy metabolism, possibly through the enhancement of mitochondria formation [53]. Similar findings were previously found in OA chondrocytes [35] but not in SNP-treated ones [10]. Therefore, an increase in mitochondrial mass could be a mechanism by which OA chondrocytes as well as NOC-12-treated cells, compensate for the electron transfer deficiency resulting from dysfunction in several complexes and the consequent low production of ATP per mitochondrion, as has already been reported by Maneiro and collaborators [35].

In relation to ATP synthesis both donors had a detrimental effect on it, but once more SNP was the compound with the most important deleterious effect. With respect to lactate production, SNP reduced the levels in a significant way compared to the control cells; on the contrary, NOC-12 increased lactate production by chondrocytes although this increment was not statistical significant. A dysfunction in complexes I, III and IV compromises the electron transfer pathway; this defect could be solved increasing the anaerobic metabolism to avoid excess production of ROS, and these findings are in agreement with the increase of lactate levels after incubation with NOC-12. These results are consistent with the findings reported by Tomita and collaborators on NOC-18-treated chondrocytes, another member of the diazeniumdiolate family [13]. Because NO inhibited the respiration of mitochondria, cellular glycolysis was enhanced significantly, the effect on cellular ATP levels was rather mild, despite the inhibition of mitochondrial respiration by NO. Thus, the enhanced glycolysis in NOC-18-treated chondrocytes could theoretically compensate for the inhibition of mitochondrial synthesis by approximately 46% [13]. On the other hand, the build-up of lactic acid will have detrimental effects on the extracellular matrix and may contribute to the pathogenesis and progression of OA [54].

Chondrocytes are highly glycolytic resident cells of articular cartilage that metabolize glucose as a primary substrate for ATP production [17]. The Pasteur effect arises in articular cartilage; in this way anaerobic glycolysis and lactate production are involved in respiratory metabolism of articular cartilage even under aerobic conditions [21,55,56]. An anaerobic metabolism could be beneficial for OA, since the products of glucose degradation (lactate and pyruvate) would act as ROS scavengers [57,58], and would assure ATP production even under conditions of mitochondrial dysfunction (defects in CRM complexes and high NO production) [35].

Only the traditional donor SNP was able to reduce glucose uptake by normal chondrocytes. Previously, we showed that the inhibition of complex IV with sodium azide modified the survival of the chondrocytes, but its effect was greater when glucose was absent. A possible explanation is that the inhibition of complex IV exclusively is not enough to induce apoptosis and other cellular events; such a reduction in the intake of glucose needs to be present to induce it [10]. The glucose dependency of chondrocytes arises with the fact that the effect of SNP on chondrocyte apoptosis correlates with glucose levels; the lower the glucose levels in the media, the highest the apoptotic levels induced.

Finally, OA chondrocytes incorporated more glucose than healthy chondrocytes under the standard experimental conditions used in this study. These findings are in consonance with a higher lactate production by OA chondrocytes than control chondrocytes (personal data). Furthermore, this up-regulation can be considered a protective mechanism that maximizes the cell's ability to capture glucose and thus to overcome stressful conditions, such as glucose scarcity or even deprivation [59,60], or just to compensate CRM defects. On the other hand, these findings can somehow explain the ROS contribution to the pathogenesis of OA [61,62]; no changes in glucose incorporation by normal chondrocytes can suggest a protective mechanism against the deleterious effects of excessive intracellular glucose, as seen in other cells [63], and the incapacity of OA chondrocytes to regulate this can trigger ROS accumulation in OA cartilage. Others authors have reported that basal glucose uptake is identical in normal and OA chondrocytes [63]; the reasons for these discrepancies are unclear but the observed differences may be related to the culture conditions used in these studies.

## Conclusions

The new generation donor NOC-12 mimics the metabolic OA situation much better than the classical NO donor SNP. Taking account of all the results obtained in this study, previous findings using SNP have to be considered very cautiously, and most of the effects observed in human chondrocytes probably cannot be attributed exclusively to NO.

## Abbreviations

CS: citrate synthase; DAPI: 49,6-dianidino-2-phenylindole dihydrochloride; 2-(^3^H)DG: 2-deoxy-(^3^H)glucose; DMEM: Dulbecco's modified Eagle's medium; FCS: fetal calf serum; IL-1β: interleukin-1β; *iNOS*: inducible nitric oxide synthase; JC-1: 5,5',6,6'- tetrachloro-1,1',3,3'-tetraethylbenzimidazole carbocyanide iodide; ΔΨm: mitochondrial membrane potential; MRC: mitochondrial respiratory chain; NO: nitric oxide; NOC-12: N-ethyl-2(1-ethyl-2 hydroxy-2-nitrosohydrazine; OA: osteoarthritis; OXPHOS: mitochondrial oxidative phosphorylation; PBS: phosphate buffered saline; ROS: reactive oxygen species; SIN-1: 3-morpholinosydnonimine; SNAP: S-nitroso-N-acetylpenicillamine; SNP: sodium nitroprusside; TNFα: tumour necrosis factor α.

## Competing interests

The authors declare that they have no competing interests.

## Authors' contributions

MCdA carried out the experimental work, analysed the data and drafted the manuscript. EM participated in the study design, interpretation of data and manuscript preparation. MAM and JA measured the MRC complex activities in digitonin-permeabilized chondrocytes. FJB conceived and coordinated the project and revised the manuscript. All the authors read and approved the manuscript.

## Supplementary Material

Additional file 1 **Table showing values of apoptotic cell death in normal chondrocytes treated with different nitric oxide (NO) donor compounds**. Click here for file

Additional file 2 Table showing values of ATP production by normal chondrocytes treated with different NO donors compounds for 24 hours*. Click here for file
