# Supplementary material for: Nitric oxide compounds have different effects profiles on human articular chondrocyte metabolism
Source: Arthritis Res Ther. 2013 Sep 11;15(5):R115. doi: 10.1186/ar4295 (PMC3978712; doi:10.1186/ar4295)
Supplement: Additional file 1 — Table showing values of apoptotic cell death in normal chondrocytes treated with different nitric oxide (NO) donor compounds. [file ar4295-S1.DOC]

**Table S1:** Values of apoptotic cell death in normal chondrocytes treated with different NO donors compounds.

|  | **12 hours** | **24 hours** | **48 hours** |
| --- | --- | --- | --- |
| Control | 0.92 ± 0.70 (5) | 1.28 ± 1.22 (7) | 0.83 ± 0.49 (9) |
| 0.5 m*M* NOC-12 | 1.84 ± 1.27 (5) | 2.82 ± 1.68 (5) | 5.83 ± 3.51 (5) |
| 1 m*M* NOC-12 | 2.47 ± 1.80 (5) | 4.26 ± 1.98 (5) | 6.01 ± 1.87 (5) |
| 2 m*M* NOC-12 | 4.83 ± 3.52 (5) | 9.63 ± 6.12 (5) | 21.59 ± 8.51 (5) |
| 0.5 m*M* SNP | 6.39 ± 8.31 (5) | 12.24 ± 18.51 (7) | 20.00 ± 27.43 (9) |
| 1 m*M* SNP | 21.13 ± 15.04 (5) | 25.86 ± 23.32 (7) | 30.82 ± 28.71 (9) |
| 2 m*M* SNP | 21.17 ± 16.46 (5) | 26.45 ± 20.40 (7) | 36.74 ± 31.39 (9) |
