# Supplementary material for: Nitric oxide compounds have different effects profiles on human articular chondrocyte metabolism
Source: Arthritis Res Ther. 2013 Sep 11;15(5):R115. doi: 10.1186/ar4295 (PMC3978712; doi:10.1186/ar4295)
Supplement: Additional file 2 — Table showing values of ATP production by normal chondrocytes treated with different NO donors compounds for 24 hours*. [file ar4295-S2.DOC]

**Table S2:** Values of ATP production by normal chondrocytes treated with different NO donors compounds for 24 hours*.

|  | **nmoles ATP (2x10^4^ cells)** |
| --- | --- |
| Control | 0.57 ± 0.19 (7) |
| 0.5 m*M* NOC-12 | 0.39 ± 0.15 (7) |
| 1 m*M* NOC-12 | 0.40 ± 0.16 (7) |
| 2 m*M* NOC-12 | 0.43 ± 0.18 (7) |
| 0.5 m*M* SNP | 0.21 ± 0.19 (7) |
| 1 m*M* SNP | 0.03 ± 0.04 (7) |
| 2 m*M* SNP | 0.01 ± 0.01 (7) |
